# Identification of Novel Modifier Genes Associated With Pain in Cystic Fibrosis: An In Silico Gene Discovery

**DOI:** 10.1155/humu/7570437

**Published:** 2025-10-19

**Authors:** Anastasia Ward, Ramil Mauleon, Chee Y. Ooi, Nedeljka Rosic

**Affiliations:** ^1^Southern Cross University, Faculty of Health, Coolangatta, Queensland, Australia; ^2^Rice Breeding Innovations, International Rice Research Institute, Los Banos, Laguna, Philippines; ^3^Southern Cross University, Faculty of Science and Engineering, Lismore, New South Wales, Australia; ^4^Discipline of Paediatrics & Child Health, Randwick Clinical Campus, School of Clinical Medicine, UNSW Medicine & Health, University of New South Wales, Sydney, Australia; ^5^Department of Gastroenterology, Sydney Children's Hospital Randwick, Sydney, New South Wales, Australia

**Keywords:** bioinformatics, cystic fibrosis, in silico, modifier genes, pain, phenotype

## Abstract

**Background:**

Cystic fibrosis (CF) is the most common life-shortening monogenic autosomal recessive disease in Caucasians with diverse and extensive comorbidities. Where the majority of studies have focused on the respiratory and digestive systems, there has been a paucity of research focusing on pain, even though people living with CF have reported a high prevalence and increased severity of pain. Many studies have identified the complex relationship between genotype and phenotype, and growing evidence suggests that the phenotypic variation observed not only depends on the variations in the CF transmembrane conductance regulator (*CFTR*) gene but also on modifier genes. Gene modifiers (GMs) have been reported to affect many organs or systems in CF. However, there have been no studies on how GMs may influence pain. Therefore, this study is aimed at highlighting potential modifier genes that may affect pain perception in CF and possible responses to therapeutics.

**Methods:**

The bioinformatics workflow adopted includes database and literature mining, pathway enrichment analysis, protein–protein interactions evaluation and drug–gene network investigation.

**Results:**

We identified seven potential pain modifiers in CF, including chymotrypsin C (*CTRC*), serine protease inhibitor Kazal-Type 1 (*SPINK1*), tumour necrosis factor (*TNF*), ATP-binding cassette subfamily B Member 1 (*ABCB1*), protease serine 1 (*PRSS1*) and transforming growth factor beta 1 (*TGFB1*) interacting with the *CFTR* gene. The analysis of the biochemical pathways indicates that signal transduction and the immune system are likely to be involved in pain processes. The specific GMs, *TNF* and *ABCB1*, are found to be within the central hub genes, indicating their potential influence on the pain pathways in CF.

**Conclusions:**

This in silico analysis highlights potential genes and biochemical pathways implicated in pain pathways that could significantly impact pain perception in people living with CF and their response to prescribed therapies. Further functional analyses are needed to include CF participants and provide a physiological relevance on how genetic polymorphisms of identified GMs may impact their pain phenotype or profile.

## 1. Introduction

Cystic fibrosis (CF) is the most common life-limiting autosomal recessive disease, affecting approximately an estimated 105,000 people globally and over 33,989 in the United States alone [1]. Attributable to over 2121 documented mutations (http://genet.sickkids.on.ca/ accessed on the 12th of October 2024) in the cystic fibrosis transmembrane conductance regulator (*CFTR*) gene, CF is a debilitating multisystem genetic disorder. The CFTR gene encodes for the CFTR transmembrane protein, an ATP-binding cassette (ABC) transporter functioning as a chloride channel located on the apical membrane of secretory epithelial cells [2]. Affecting multiple organs, primarily the respiratory, digestive and reproductive systems, the disease is characterised by physiological symptoms such as recurrent lung infections causing bronchiectasis, CF-related diabetes, endocrine and exocrine pancreatic insufficiency, infertility, chronic hepatobiliary disease, gastrointestinal obstruction, malnutrition and psychological symptoms, including anxiety and depression [3].

In addition to the hallmark characteristics of CF, recent research has highlighted the significance of pain in people with cystic fibrosis (pwCF) [4]. The International Association for the Study of Pain (IASP) defines pain as an unpleasant sensory and emotional experience associated with actual or potential tissue damage or described in terms of such damage. Pain, in general, is highly personal and extremely subjective and is a complex area of research influenced by dynamic multidirectional interactions between social, psychological and biological factors [5], similar to the complex phenotypes and clinical heterogeneity of CF.

In the last 20 years, significant empirical attention has been paid to genetic contributions to the pain profile. This has led to numerous novel findings, including how genetic variations influence pain perception, the responses to pain management therapies, susceptibility to chronic pain conditions and identifying the specific biological pathways that may contribute to the pain profile [5]. However, the mechanisms of pain in CF, its associations with other symptoms and how pain changes across the lifespan are not well understood and have yet to be explored [6]. Extensive research investigating gene modifiers (GMs) or additional genetic factors that influence the disease phenotype [7–10] outlines the importance of searching for potential candidate genes acting upon the CF phenotype, particularly in pain.

Ever-emerging bioinformatics tools, computational algorithms and now artificial intelligence play a critical role in decoding vast quantities of genomic data and identifying candidate genes of interest [11, 12]. In silico methods, or data mining, organising, analysing and predicting pathogenicity in a silicon substrate, are instrumental when analysing complex biochemical pathways or protein networks [11, 13–15]. Particularly, they can highlight the upstream underlying molecular mechanisms of the disease phenotypes or symptoms that may influence protein expression. Utilising in silico methods and high-throughput quantification of genomic variations continues to break new ground in the pursuit of precision medicine [16, 17]. Previous studies have successfully used in silico protocols to uncover potential biomarkers and signalling pathways in various health conditions such as cancer [18] and CF [19, 20]. However, there have been no studies, either in silico or functional, evaluating the underlying mechanisms that potentially contribute to pain in CF [9]. By conducting an in silico gene discovery study, we aim to assess genes implicated in the pain pathways, examine their functional relationships with the CFTR gene and potentially highlight novel genes, hub genes or pathways that may influence the pain profile in CF. Identifying potential GMs of pain will not only enhance our understanding of pain mechanisms in CF but also contribute to the development of biomarker-driven diagnostics and therapeutic strategies in CF [16]. Further, it will also contribute to the literature surrounding the effects of modulator therapies on pain, whereby research has highlighted that modulator therapies such as ivacaftor, lumacaftor, tezacaftor and elexacaftor have no effect on pain in CF [21].

## 2. Methods

### 2.1. Data Sources and Gene Selection

The selection of genes was conducted using an extended methodology previously performed by Lipner et al. [13] and Trouve et al. [15]. Genes associated with CF and pain were identified using four publicly available databases, which included the Online Inheritance in Man (OMIM) database, the Human Genome Epidemiology encyclopedia (HuGE Navigator) using the phenopedia function, the Phenotype-Genotype Integrator (PhenGenI) and the Comparative Toxicogenomics Database (CTD). OMIM is regarded as the best curated, most comprehensive and most authoritative resource of genotype-phenotype relationships [22]; CTD is a unique database that curates relationships between specific genes and human diseases by integrating chemical and protein–disease relationships; HuGE Navigator is a comprehensive database of population-based epidemiological studies of human genes, and PhenGenI is a web-based platform that integrates National Center for Biotechnology Information (NCBI) data with genome-wide association data catalogued by the National Human Genome Research Institute (NHGRI). The four databases were selected due to the breadth of information available and the unique approach to classifying human disease–gene associations. Venn diagrams were created to visually describe the genes collated and those commonly reported across the databases.

Pain in the CF keyword search

i. For searches in OMIM, keywords consisted of “cystic fibrosis”, “CF-associated genes” and “pain”.
ii. For searches in the Comparative Toxicogenomics Database, “cystic fibrosis and pain” were utilised.
iii. HuGE Navigator and PhenGenI keywords also included “pain” and “cystic fibrosis”.

### 2.2. Comparison of Genes With Published Data

Known GMs in CF with functional studies were retrieved from works published in PubMed and a current systematic review of GMs in CF [9]. Further, a systematic review of pain [23] was also utilised, and genes demonstrated to be associated with pain were extracted. Finally, the Human Pain Genetics Database, a comprehensive inventory of genetic contributors to pain, was also utilised. All genes in the database were downloaded into an Excel table. Another Venn diagram was drawn from the three sources to identify commonly expressed genes.

### 2.3. Expression Analysis of Genes

The expression analysis of genes in CF and pain was collected from the Gene Expression Omnibus (GEO) database (https://www.ncbi.nlm.nih.gov/geo/ (accessed on the 22^nd^ of January 2024)) and passed through the corresponding GEO2R (https://www.ncbi.nlm.nih.gov/geo/geo2r/) application for the analysis. The experimental groups in both datasets were reassigned for this study. The dataset for the CF analysis included GSE40445 (gene expression in CF-versus-non-CF airway epithelial cells from nasal brushing). The dataset was selected due to its focus on the p.Phe508del mutation, the most common CF genotype and tissue specificity focusing on the lungs or airway epithelium, which is the most studied location of GMs [9]. The focus on airway epithelium—tissue directly involved in inflammation, a hallmark feature of CF [24] and a recognised contributor to pain in the general population [25], allows the identification of potentially relevant inflammatory markers.

Due to the nature of pain and different types or locations identified in a previous pain-related study in CF [21], three datasets, including GSE127208 (towards precision medicine for pain: diagnostic biomarkers and repurposed drugs), GSE24982 (mRNA expression profiling in the spinal nerve ligation model of neuropathic pain in rats) and GSE176223 (identification of synovial fibroblast subsets associated with pain and progression of knee osteoarthritis) were included for pain. The GEO2 interactive web application utilises the limma (Linear Models for Microarray Data) package. It comprehensively analyses complex investigations to identify genes differentially expressed according to certain conditions. The *Benjamini* and *Hochberg false discovery rate* method was utilised as described in the methodology performed by Trouvé et al. [15]. Genes of interest were then selected using the Bonferroni-corrected *p* value of 0.05. Differentially expressed genes were then recorded and included in the Venn diagram.

### 2.4. Biochemical Pathway Analysis

Target genes were then mapped to biological pathways using the Venn Viewer function in CTD. Venn Viewer's pathway analysis integrates data from the Kyoto Encyclopedia of Genes and Genomes (KEGG), REACTOME and BioGRID to find molecular mechanisms underlying disease processes and potential toxicological responses. The parameters were set to the Venn Viewer pathways associations function. The KEGG pathways and REACTOME pathways were also downloaded.

### 2.5. Gene–Disease Associations

CTD was also utilised to perform a gene–disease association analysis. Associations are extracted from the OMIM database using a mim2gene file from the NCBI database or the peer-reviewed published literature. CTD biocurators manually curate gene–disease associations and pathways from the literature.

### 2.6. Protein–Protein Network Exploration

Protein–protein interactions (PPIs) are critical in many biological processes, particularly signal transduction, drug development and understanding cellular functions in various metabolic pathways [26]. To explore the PPIs and networks of the potential GMs, STRING 12.0 (Search Tool for the Retrieval of Interacting Genes and Proteins (https://string-db.org/)) and GeneMANIA (GeneMANIA) were utilised. STRING, a comprehensive global resource currently containing data on 59,309,604 proteins and 12,535 organisms, is used to search for connections or edges between proteins (nodes) or genes. The tool parameters were set to a medium confidence level of 0.400 and *Homo sapiens* to retrieve a wide range of potentially interacting genes.

Gene MANIA was then explored using the same potential genes so that, available through the integrated Cytoscape application, a network visualisation could be created and analysed. Furthermore, GeneMANIA provided a ranking of the interacting proteins based on the strength and type of the association, allowing for the identification of potential hub genes or genes that have a high number of interactions with other genes in the gene network [27]. Hub genes are often investigated as dysfunctional hub genes can have widespread effects and are often associated with disease [27].

### 2.7. Drug–Gene Interactions

Finally, the Drug–Gene Interaction Database (DGIdb) was used to identify interacting drugs targeting the potential modifiers.

## 3. Results

### 3.1. Study Design

**Figure 1:**
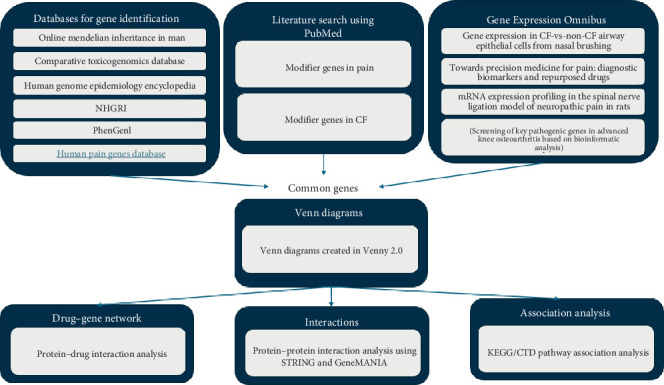
Workflow of the methodology employed utilising the keywords “pain” and “cystic fibrosis”.

The workflow of our methodology is described in Figure 1. Genes were retrieved from databases, including OMIM, HuGE Navigator, PhenGenI and CTD. The literature was also scanned to identify differentially expressed genes, and datasets from GEO were also identified for the analysis. Candidate genes reported in the databases were compared and analysed for comparable biochemical pathways or genes. Further, PPIs were also investigated to provide a physiological relevance between the investigated genes and genes of interest.

### 3.2. Gene Selection

**Figure 2:**
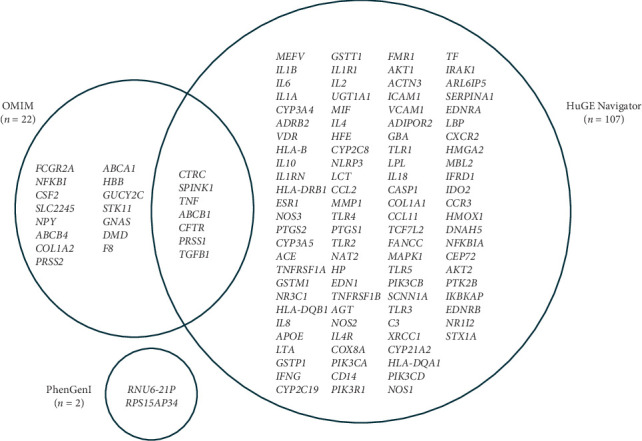
Common genes reported in the following databases: OMIM, HuGE Navigator, PhenGenI, NIH and CTD.

Utilising the keywords “cystic fibrosis” and pain in OMIM, 125 and 734 genes were reported as described in Table 1, respectively. Then, 22 genes were common to both pain and CF in OMIM. CTD reported 13 genes in CF and 91 in pain, with no genes overlapping. HuGE described 352 genes significant in CF and 967 in pain. The intersection depicted 107 overlapping genes in both datasets. PhenGenI reported 14 in CF and 18 in pain, whereby there were two common genes. The NHGRI database retrieved 12 genes in CF and 20 in pain, whereby there were no common genes. A total of 1516 and 1830 were retrieved for CF and pain. When comparing the common genes across all five databases, as described in Figure 2, seven genes were reported in multiple databases, including chymotrypsin C (*CTRC*), serine protease inhibitor Kazal-Type 1 (*SPINK1*), *TNF*, ATP-binding cassette subfamily B member 1 (*ABCB1*), *CFTR*, *PRSS1* and transforming growth factor beta 1 (*TGFB1*).

### 3.3. Known Modifier Genes in CF and Published Work on Pain

**Figure 3:**
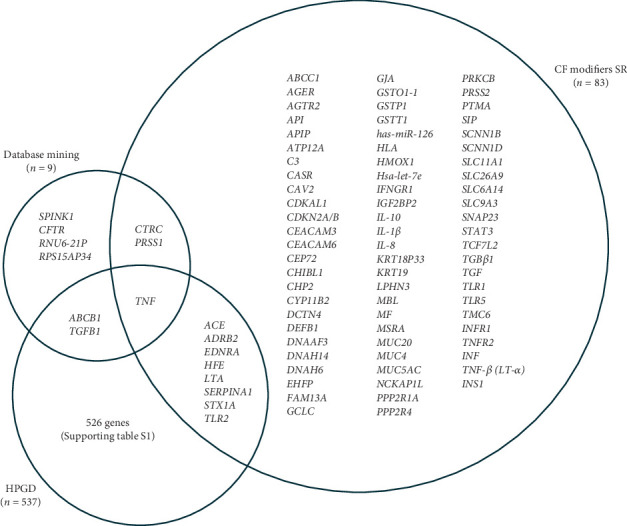
Common genes reported in the initial databases, the Human Pain Genes Database and a literature scan of known CF gene modifiers' systematic review (SR).

A literature scan from the last 14 years identified 83 known modifiers of CF and 41 modifiers of pain. When identifying genes, the Human Pain Genes Database reported 537 genes that have been implicated in affecting pain. Eight known CF modifier genes, including *ACE*, *ADRB2*, *EDNRA*, *HFE*, *LTA*, *SERPINA1*, *STX1A* and *TLR2,* were commonly reported in the CF-specific systematic review [9] and in the HPGD. Two genes, *CTRC* and *PRSS1*, were common in the CF-specific systematic review [9] and the genes retrieved from the initial five databases. Two genes were common to the five databases and the HPGD. Finally, one gene, *TNF*, was common in all three data mining exercises, as described in Figure 3.

### 3.4. Differential Gene Expressions

Based on the retrieval of the four gene array datasets from GEO, GSE40445 (gene expression in CF-versus-non-CF airway epithelial cells from nasal brushing), GSE127208 (towards precision medicine for pain: diagnostic biomarkers and repurposed drugs), GSE24982 (mRNA expression profiling in the spinal nerve ligation model of neuropathic pain in rats) and GSE176223 (screening of key pathogenic genes in advanced knee osteoarthritis based on bioinformatic analysis), a total of 394 genes were identified. Of these genes, 214 were retrieved from GSE40445, four from GSE176223, 116 from GSE24982 and 60 from GSE127208, as described in Table S1. Of the three pain datasets, no genes were expressed in another subset. Further, when the CF dataset was added, again, no genes were commonly expressed.

### 3.5. Analysis of Biochemical Pathways

**Figure 4:**
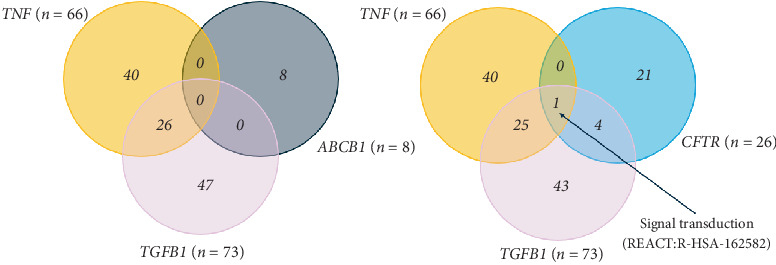
Biochemical pathways association of potential modifier genes in KEGG and REACTOME using Venn Viewer.

The target genes *TNF*, *ABCB1* and *TGFB1*, described from the intersection of the genes retrieved from the five databases, known CF GMs and the HPGD, were then mapped to their biological process pathways using the Venn Viewer function in CTD (https://ctdbase.org/tools/vennViewer/).  Table 2 describes the pathways involved in each gene and is also described in Table S2. Only TNF and TGFB1 had intersecting pathways, whereby 26 pathways were described. The KEGG and REACTOME ID numbers are also reported in Table 2. Due to intersecting pathways, TNF, TGFB1 and the CFTR gene were also analysed to understand the pathways specifically in CF that may be impacted, as described in Figure 4. The pathway common to all three genes is signal transduction.

### 3.6. PPI Networks and the Biological Function of Candidate Genes

**Figure 5:**
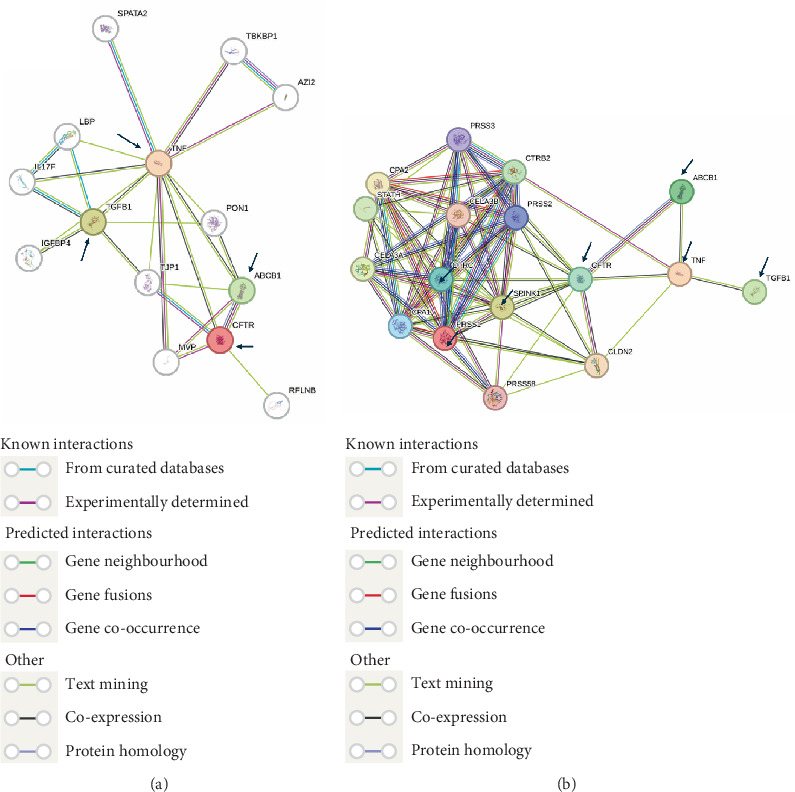
PPI networks as described by STRING.

**Figure 6:**
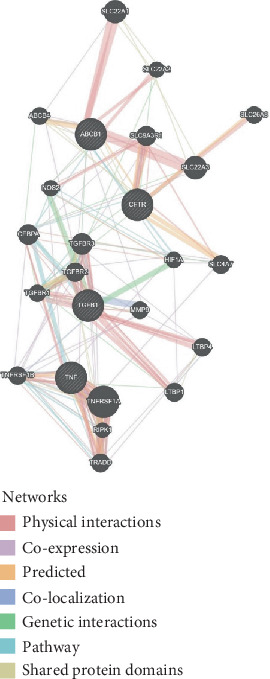
PPI networks as described by GeneMANIA.

**Figure 7:**
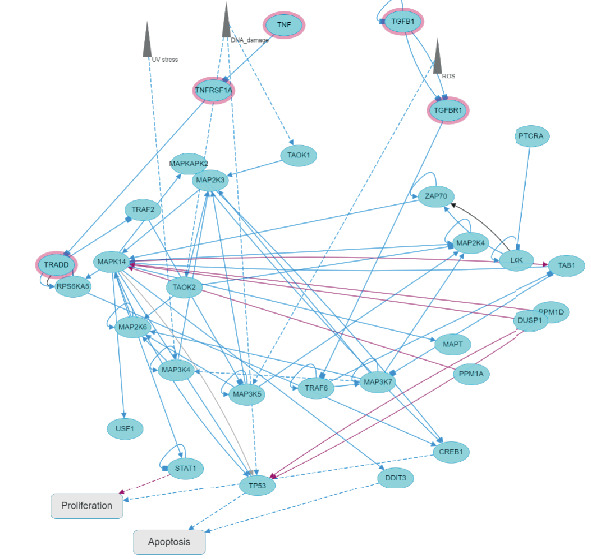
P38 pathways and the role of the potential gene modifiers.

The exploration of the interactions between the proteins TNF, CFTR, TGFB1 and ABCB1 was conducted in STRING Version 12.0 (https://string-db.org/), which consisted of 14 nodes and 26 edges, as described in Table 3. In Figure 5, the protein list for PPI was expanded to CTRC, SPINK1, TNF, ABCB1, PRSS1, TGFB1 and CFTR, which consisted of 17 nodes and 73 edges. When determining the rank of importance of the interacting proteins, GeneMania reported, as described in Figure 6, that tumour necrosis factor receptor superfamily member 1A (TNFRSF1A) was the most highly ranked, as described in Table 4.  Figure 7 describes the P38 pathways and the role of the potential GMs.

### 3.7. Drug–Gene Network Analysis

Utilising the DGIdb, *ABCB1* directly interacts with nine separate pain management therapeutics, *CFTR*, *TGFB1* and *TNF*, each interacting with one drug as described in Table 5.

## 4. Discussion

The pathophysiology and aetiology of pain and CF are heterogeneous and complex. Numerous studies identified a connection between pain and CF [4, 6, 28–30] with a continuous call to action from pwCF to prioritise pain in research [21, 31, 32]. However, this is the first genomic study on pain in CF that identifies potential gene biomarkers of pain. Genetic studies of pain, in general, are highly complex and complicated due to the heterogeneity of pain phenotypes, and in CF, this is also evident [21]. Here, we investigated the link between GMs of pain in CF using bibliographic mining, gene expression analysis and the evaluation of biochemical pathways to determine potential biomarkers of pain in CF.

By identifying genes associated with both pain and nonpain phenotypes in CF, gene candidates with potential dual relevance are prioritised as they may contribute to the CF pathophysiology and potentially modulate or influence pain perception. Whilst overlap does not confirm causality, it provides a starting point for gene prioritisation and future investigations. Further, using an integrative strategy, genes that intersect with known pain-associated genes may be investigated for their influence on downstream processes or the molecular mechanism that occurs after a gene has been transcribed.

Based on the database search results, seven genes of interest, including *SPINK1, CTRC, PRSS1, ABCB1, TGFB1* and *TNF* interacting with the *CFTR* gene, were identified as potentially important in the CF pain profile (Table 3).

### 4.1. *TNF*

The *TNF* gene, or *TNF-α,* encodes a multifunctional proinflammatory cytokine, a major regulator of the inflammatory response [33]. Found at high concentrations and a known biomarker of CF, *TNF* is the most studied GM in CF [9, 34], associated with an increased risk of osteoporosis, increased liver disease and cirrhosis development, increased gastrointestinal complications and also an increase in pulmonary disease severity with increased airway colonisation and levels of neutrophil elastase [9]. However, no studies have evaluated *TNF* as a GM of pain in CF. As described in the pain literature, it is a critical cytokine that contributes to the sensitisation of both the peripheral and central pain pathways, enhancing pain sensitivity leading to hyperalgesia or increased sensitivity to pain or allodynia, the feeling of pain where there are no pain stimuli [35]. In a study conducted by Inglis et al. [36], anti-TNF therapy was observed to produce rapid analgaesia and suggested that under noninflammatory conditions, it acted on peripheral cells to induce a proinflammatory cascade resulting in increased sensitisation and activation of nociceptive neurons. Further, mutations or variations in the *TNFRSF1A* gene, responsible for binding TNF, are associated with tumour necrosis factor receptor-associated autoinflammatory syndrome (TRAPS), characterised by fever, limb and abdominal pain [37].

### 4.2. *SPINK1*, *PRSS1* and *CTRC*

In the pancreas, CFTR plays a critical role in the regulation of chloride and bicarbonate ions. In CF, this balance is impaired and causes progressive ductal or glandular obstruction and inflammation due to the acidic and viscous pancreatic secretions, causing pancreatitis [38, 39]. Pancreatitis is characterised by pain, and it is often a key diagnostic feature of the disease [40]. The gene identification methods employed in this study detected several genes, *SPINK1*, *PRSS1* and *CTRC,* that are well-known GMs of pancreatitis in CF [9, 41].

*SPINK1* is a trypsin kinase inhibitor, predominantly expressed in the pancreas and gastrointestinal tract, known to regulate the nuclear factor erythroid 2-related factor 2 (Nrf2) pathway, an antagonist of the NF-*κ*B pathway [42]. Therefore, under conditions of oxidative stress, SPINK1 is an antioxidant promoter. In CF, SPINK1 is often upregulated and, depending on any mutations in the gene, can influence the progression of several diseases, specifically pancreatitis in CF and pancreatic cancer [43]. However, there is evidence that the Nrf2 pathway is already impaired in cells with mutated CFTR [44].

The serine protease 1 (*PRSS1*) gene encodes for the enzyme cationic trypsinogen, a precursor for trypsin, the most abundantly digested enzyme produced in the pancreas [45]. Trypsinogen and chymotrypsin are the most studied zymogens or proenzymes and are shown to have significant roles in tissue regeneration, depicting an anti-inflammatory effect, antioxidant, anti-infective, anti-edematous and fibrinolytic [46].

The *CTRC* gene encodes for an enzyme that regulates the activation and promotes proteolytic inactivation of digestive enzymes such as trypsinogen and trypsin [47]. In CF, *CFTR* gene mutations facilitate trypsinogen activation due to decreased ductal flushing and altered intraductal pH levels. In CF, the *CTRC* gene protects against harmful trypsinogen activation, protecting against pancreatitis. Pathogenic mutations or variants in the *CTRC* gene cause loss of function, affecting secretion and proteolytic stability, and secretion increases the risk of chronic pancreatitis, whereby oxidative stress is a major contributor to the inflammation and fibrosis observed in the pancreas [47]. Pathogenic mutations in both the *CTRC* and *PRSS1* genes have been associated with pancreatitis, where oxidative stress significantly contributes to inflammation in the pancreas and exacerbates pain pathways [47].

### 4.3. *TGFB1*

*TGFB1* is a gene critical in regulating cell functionality such as cell growth and differentiation, apoptosis, immune system regulation and the formation of fibrosis [48]. In CF, *TGFB1* is activated by bacterial infection, malabsorption or nutritional status and also by polymorphisms in the *TGFB1* gene itself [49]. It is a significant contributor to the inflammatory process and the development of lung fibrosis, specifically inhibiting the degradation of the extracellular matrix and promoting smooth muscle cell hypertrophy and hyperplasia [50]. Further, it also prevents the biogenesis of the CFTR protein, impairing the functional rescue of the p.Phe508del mutation CFTR and potentially compromising the efficacy of CFTR therapies or correctors [49]. Regarding pain, *TGFB1*, as described in the results, activates the mitogen-activated protein kinase (MAPK) pathways, or known pathways, and has also been shown to increase ROS production, inducing a redox imbalance or oxidative stress [51]. This redox imbalance and activation of the MAPK pathways influence the overall fibrosis in CF and potentially contribute to pain sensitisation. Genomic variations in the *TGFB1* gene cause Camurati–Engelmann disease (CED), which influences the thickening of the long bones' diaphysis and presents with symptoms including bone pain, muscle weakness and fatigue [52]. To date, there have been no studies on mutations in the *TGFB1* gene and pain in CF.

### 4.4. *ABCB1*

The *ABCB1* is a member of the superfamily ABC transporters and encodes a transmembrane ATP-dependent drug efflux pump that is critical in protecting cells and transporting various drugs across cell membranes, particularly along the blood–brain barrier [53, 54]. Due to the functionality of the efflux pump and drug resistance, the *ABCB1* gene has been identified as a potential therapeutic target [55]. Coluzzi et al. [53] reported that increased activity of ABCB1 leads to reduced activity of certain pain medications, diminishing their analgesic effects. In pwCF, inflammatory conditions, such as CF and prolonged xenobiotic exposure, are hypothesised to upregulate ABCB1. Polymorphisms or variations in the *ABCB1* gene, altering absorption, distribution and excretion, have been associated with altered phenotypes or differences in pain perception [53]. To date, no studies have investigated the effects of genetic variations in the *ABCB1* gene on the clinical symptoms of CF. As described in the results, the *ABCB1* interacted with nine pain management therapies, including acetaminophen, aspirin, capsaicin, codeine, gabapentin, ibuprofen, methadone, naproxen and pentazocine, which have been reported to be utilised by pwCF. Specifically, a significant difference was reported in the efficacy of nonsteroidal anti-inflammatory drugs (NSAIDs), which could be associated with *ABCB1* genetic polymorphisms. *ABCB1* is described as the main molecular cause of clinical drug failure and should be investigated further in pain studies in CF.

Due to the intersection of *ABCB1*, *TGFB1* and *TNF* with genes in the HPGD, these three genes were carried forward to analyse their biochemical pathways and interactions with pain management strategies to identify other interacting proteins of interest. Over 120 curated pathways were identified, as described in Figure 4 and Table 2, whereby signal transduction was the most prevalent pathway. Signal transduction is the conversion of physical or chemical stimuli whereby somatosensory processes assist in opening ion-gated channels or an electrochemical signal perceived by higher order nervous centres [56]. In particular, the most commonly reported gene across the databases, *TNF*, is involved in the signal transduction of pathways, including the nuclear factor kappa-light-chain-enhancer of activated B cells (NF-*κ*B) pathway and MAPK pathways [35]. The MAPK pathways are signalling pathways that regulate cellular activities, including growth, division and differentiation [57]. In nociceptive neurons, activation of the MAPK pathway is known to influence the expression of genes involved in pain signalling and contributes to pain hypersensitivity and prolonged pain [58]. Targeting this signalling pathway could be a promising approach when developing novel analgesics.

The NF-*κ*B pathway is significant in CF as it plays a critical role in the inflammatory response, controlling cytokine production and cell survival [59]. NF-*κ*B overexpression is intensified by the hyperproduction of reactive oxygen species (ROS) and, in turn, exacerbated by bacterial stimulation, producing an increase of oxidising molecules and cytokines such as TNF, interferon-gamma (IFN-*γ*), IL-1, IL-6 and IL-17A [60]. There is extensive evidence outlining the role of oxidative stress and the progression of lung disease in pwCF in the literature [60]. Oxidative stress is the term used to describe the process or imbalance of ROS and the natural physiological defence mechanism—the antioxidant system. In pwCF, there is a sustained redox imbalance due to several factors, including malabsorption of dietary antioxidants, the excessive release of oxidants by neutrophils and the inability to efflux glutathione by the CFTR protein [61]. In the pain literature, it is hypothesised that oxidative stress influences the intensity of pain by increasing inflammation and neuropathy [58]. All of the genes described in the results contribute to oxidation, particularly *TNF*. Unravelling the key genes and molecular pathways involved in pain and CF, particularly oxidative stress and the NF-*κ*B and MAPK pathways, this study provides the first steps for future research and the development of novel pain targets.

## 5. Limitations

A major limitation of this study is the confines of the datasets themselves. Microarray gene lists, in particular, have previously been identified to demonstrate substantial variability across experimental conditions and tissues [62, 63]. In the future, broader validation across multiple datasets may improve generalisability and mitigate bias. Further, this study also does not consider the dynamic changes in gene expression, particularly in CF and pain. Lifelong treatment of CF and disease progression can contribute to dynamic changes in gene expression, which are critical for future studies to evaluate. Finally, although this study proposes potential biomarkers of pain in CF, clinical samples in conjunction with continuous self-reported pain questionnaires will need to be investigated.

## 6. Conclusion

Due to the lack of clinical evidence and appropriate functional studies on GMs impacting the pain pathways in CF, the presented *in silico* approach provides a starting point for mapping future functional studies related to pain and *genetic disease modifiers.* Here, utilising comprehensive bioinformatics methods, seven genes were identified with potential roles in pain perception and therapeutic interactions. Regulatory network and pathway analyses were constructed for known GMs involved in pain that indicated interactions with the CFTR protein. Consequently, molecular network analyses suggest potential implications of these GM genes on the pain pathways and possible importance for consideration in CF pain management strategies. Moreover, we have proposed an *in silico* methodology that is intended to generate testable hypotheses and should be used as a first-line exploratory approach when investigating pain in CF and other diseases that depict extreme phenotypic heterogeneity.

## Figures and Tables

**Table 1:** The number of genes reported in the databases, including OMIM, HuGE Navigator, PhenGenI, NIH and CTD.

**Database**	**OMIM**	**CTD**	**HuGE**	**PhengenI**	**NIH**
CF	125	13	352	14	12
Pain	734	91	967	18	20
Number of common genes to both searches in the one database	22	0	107	2	0

**Table 2:** Biochemical pathways association of potential modifier genes in KEGG and REACTOME related to known pain pathways.

**Gene**	**Chemical pathway**	**KEGG/REACTOME ID**
*TNF only*	Amyotrophic lateral sclerosis (ALS)	KEGG:hsa05014
	Death receptor signalling	REACT:R-HSA-73887
	IL-17 signalling pathway	KEGG:hsa04657
	NF-kappa B signalling pathway	KEGG:hsa04064
	NOD-like receptor signalling pathway	KEGG:hsa04621
	Regulation of TNFR1 signalling	REACT:R-HSA-5357905
	TNF signalling	REACT:R-HSA-75893
	TNF signalling pathway	KEGG:hsa04668
	TNFR1-induced NFkappaB signalling pathway	REACT:R-HSA-5357956
	TNFR1-induced proapoptotic signalling	REACT:R-HSA-5357786
	TNFR1-mediated ceramide production	REACT:R-HSA-5626978
	TNFR2 non-canonical NF-*κ*B pathway	REACT:R-HSA-5668541
	Toll-like receptor signalling pathway	KEGG:hsa04620

*ABCB1 only*	ABC transporters	KEGG:hsa02010
	ABC-family protein–mediated transport	REACT:R-HSA-382556
	MicroRNAs in cancer	KEGG:hsa05206

*TGFB1 only*	FoxO signalling pathway	KEGG:hsa04068
	Influenza virus-induced apoptosis	REACT:R-HSA-168277
	TGF-beta signaling	KEGG:hsa_M00680
	Th17 cell differentiation	KEGG:hsa04659

*TNF and TGFB1 intersect*	AGE-RAGE signalling pathway in diabetic complications	KEGG:hsa04933
	Cytokine signaling in the immune system	REACT:R-HSA-1280215
	Cytokine–cytokine receptor interaction	KEGG:hsa04060
	Inflammatory bowel disease (IBD)	KEGG:hsa05321
	MAPK signalling pathway	KEGG:hsa04010
	Rheumatoid arthritis	KEGG:hsa05323
	Signal transduction	REACT:R-HSA-162582
	TGF-beta signalling pathway	KEGG:hsa04350

**Table 3:** Protein–protein interaction pathways of identified candidate genes.

	**Gene**	**Number of nodes**	**Edges**	**PPI enrichment ** **p** ** value**	**Protein–protein interaction**
*1*	*ABCB1*	11	27	1.05e − 05	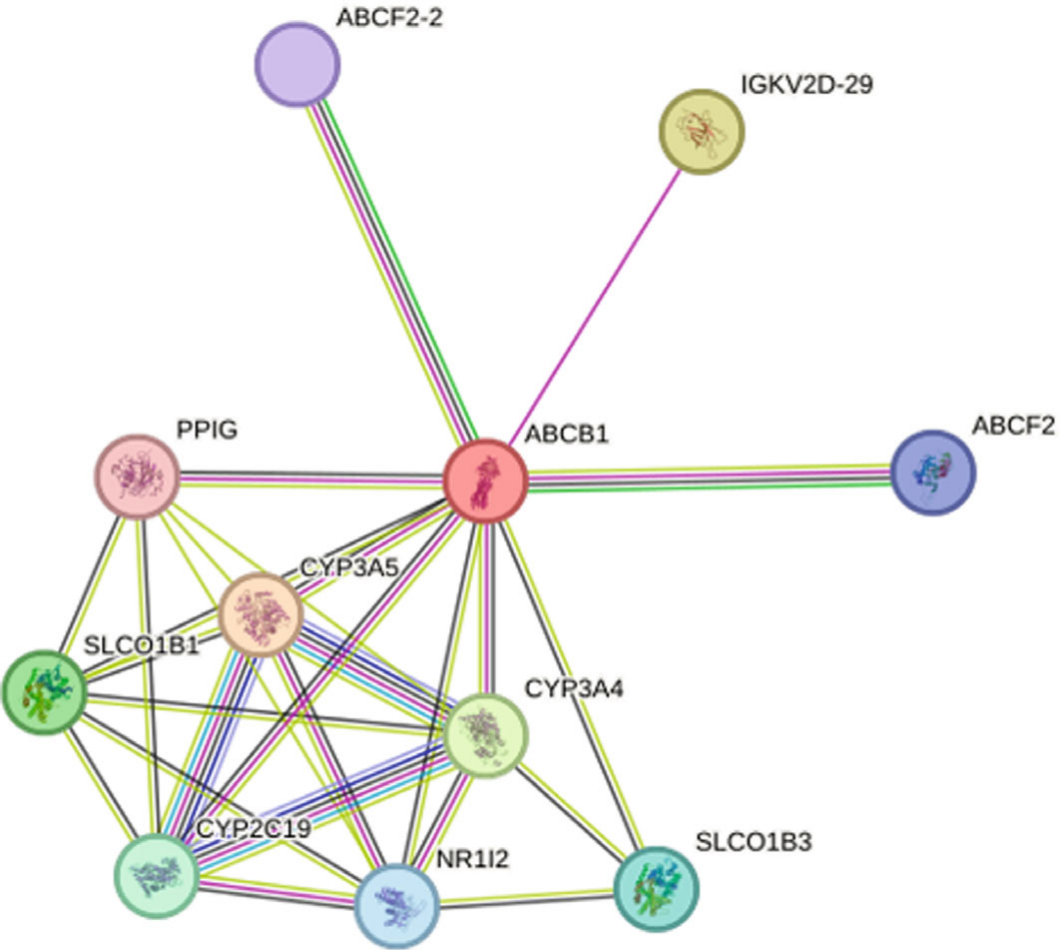
*2*	*SPINK1*	11	23	0.000311	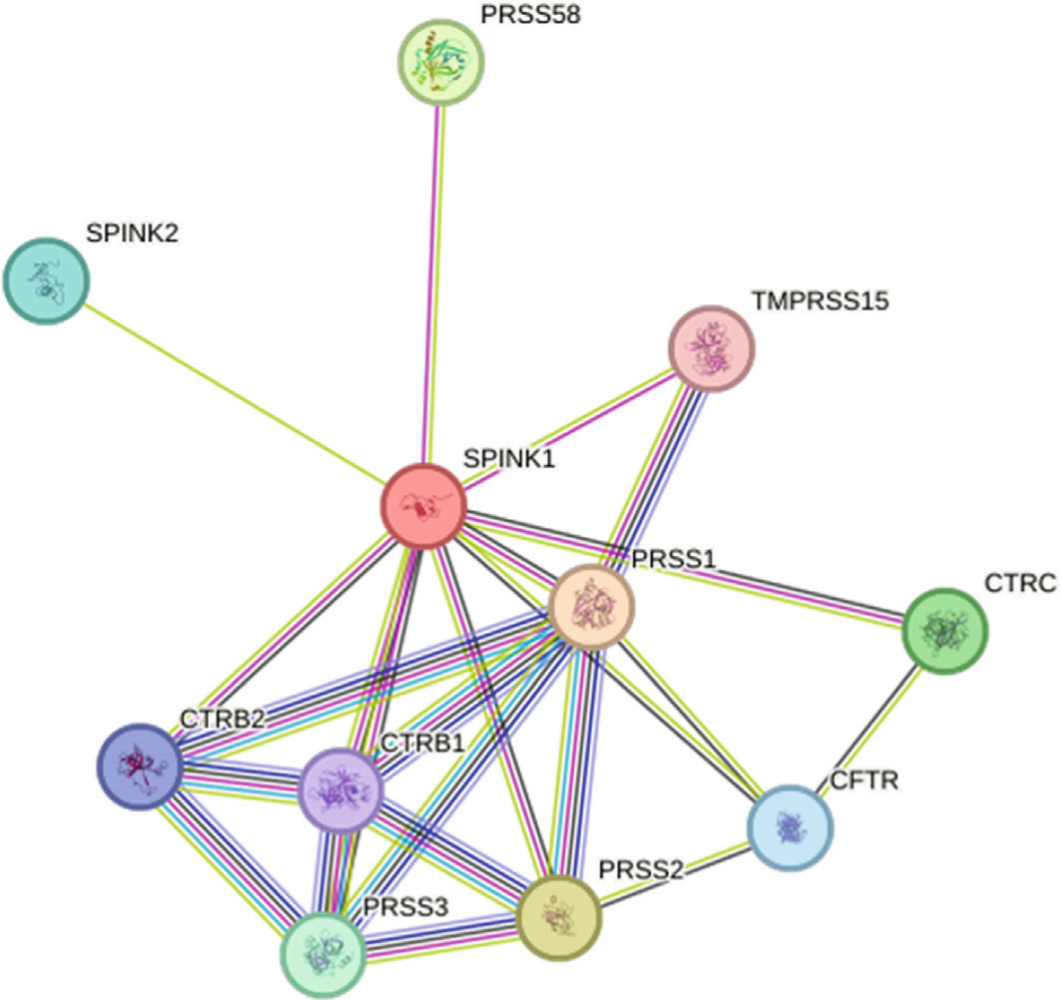
*3*	*PRSS1*	11	24	0.000175	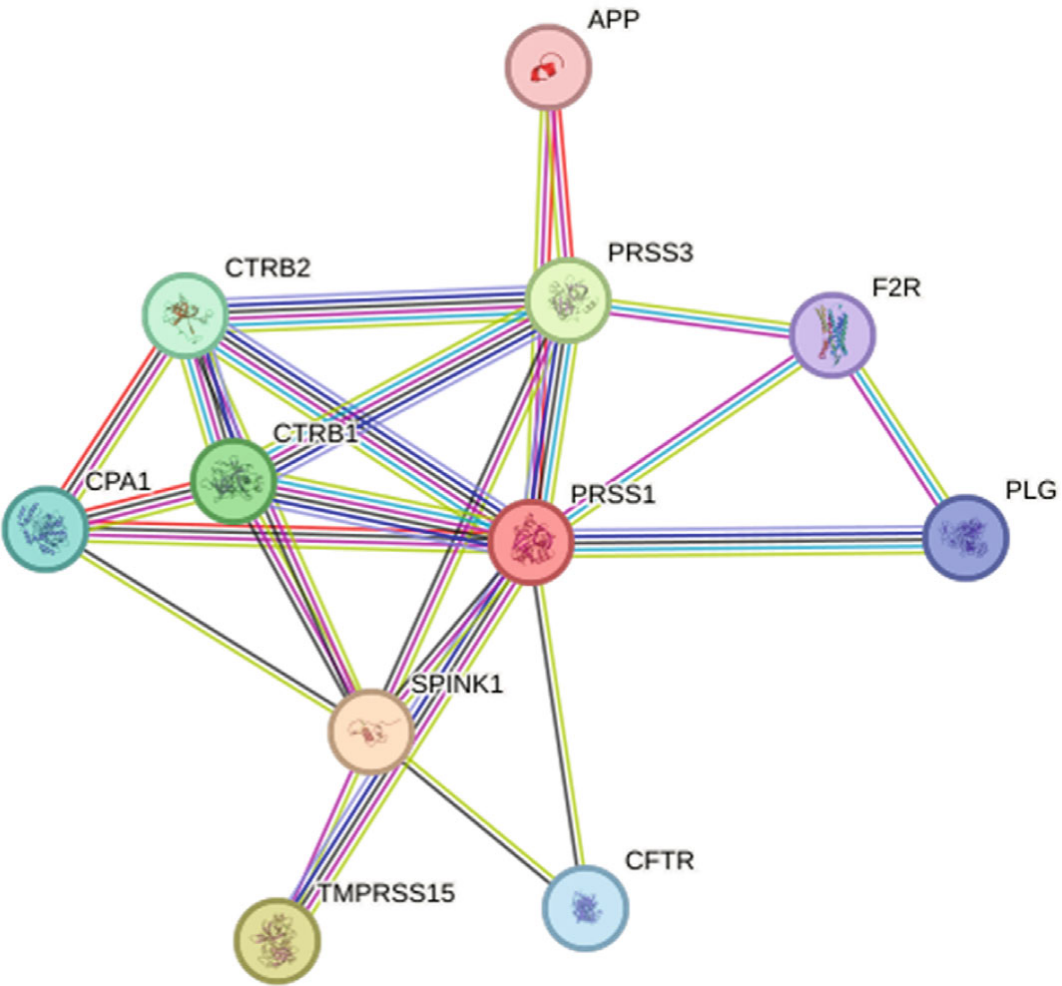
*4*	*CTRC*	11	31	9.22e − 08	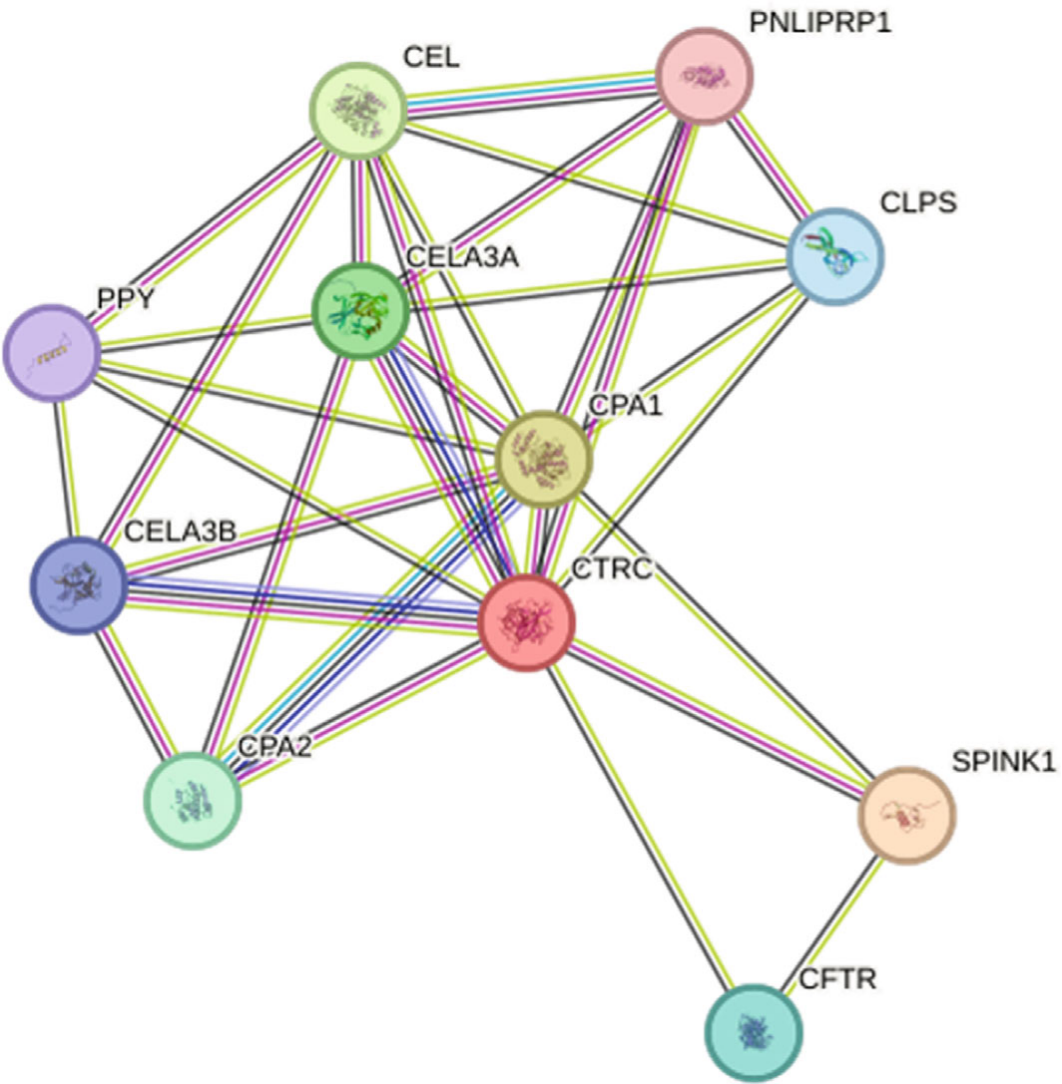
*5*	*TGFB1*	11	17	0.0346	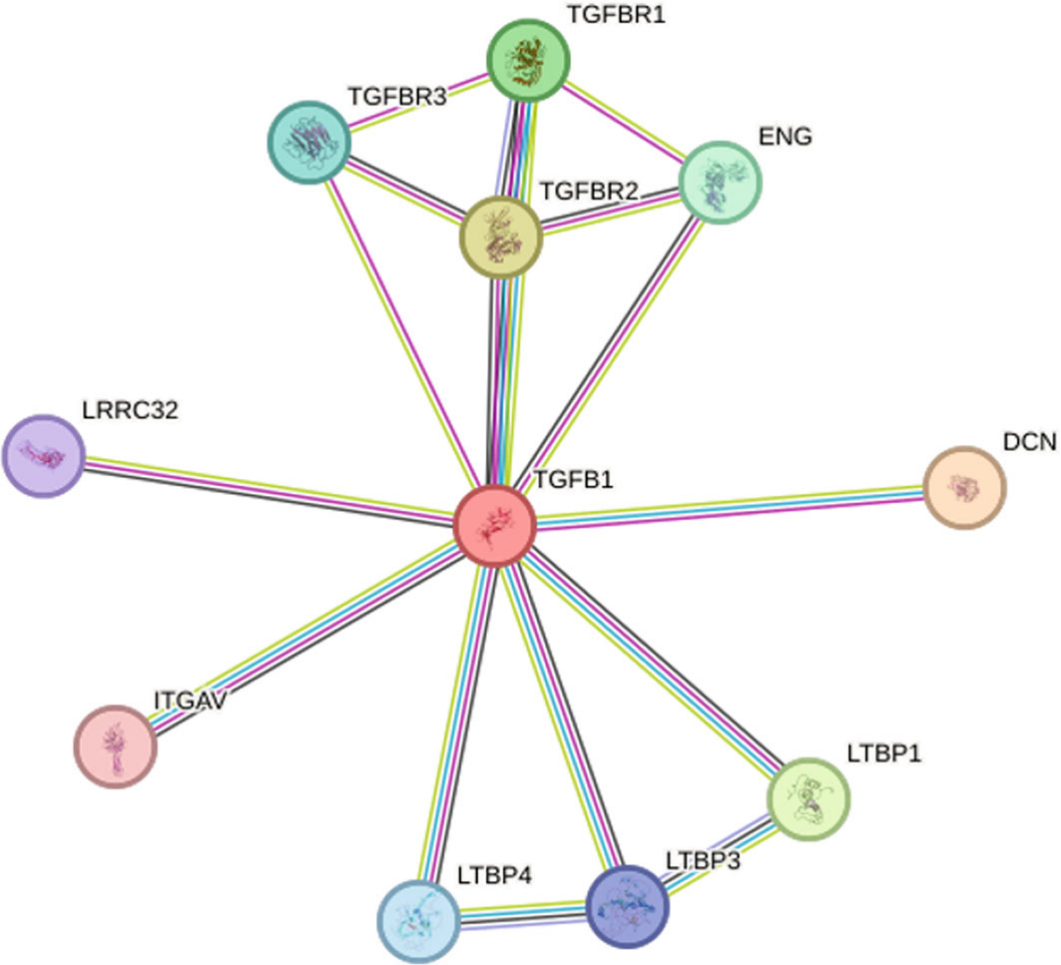
*6*	*TNF*	11	48	5.55e − 15	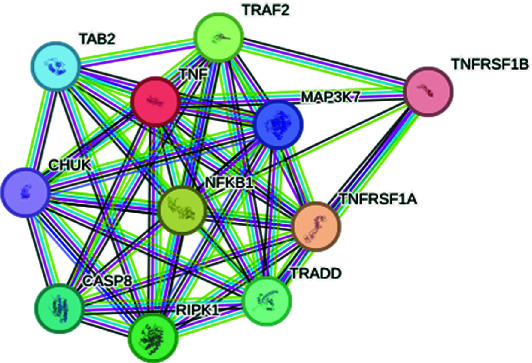

**Table 4:** Rank of importance of the interacting proteins, as reported by GeneMania.

**Gene**	**Description**	**Rank**
*ABCB1*	ATP-binding cassette subfamily B member 1 (source: HGNC symbol; Acc: HGNC:40)	N/A
*CFTR*	CF transmembrane conductance regulator (source: HGNC symbol; Acc: HGNC:1884)	N/A
*TGFB1*	Transforming growth factor-beta 1 (source: HGNC symbol; Acc: HGNC:11766)	N/A
*TNF*	Tumor necrosis factor (source: HGNC symbol; Acc: HGNC:11892)	N/A
*TNFRSF1A*	TNF receptor superfamily member 1A (source: HGNC symbol; Acc: HGNC:11916)	1
*SLC22A3*	Solute carrier family 22 member 3 (source: HGNC symbol; Acc: HGNC:10967)	2
*SLC22A1*	Solute carrier family 22 member 1 (source: HGNC symbol; Acc: HGNC:10963)	3
*TGFBR3*	Transforming growth factor beta receptor 3 (source: HGNC symbol; Acc: HGNC:11774)	4
*CEBPA*	CCAAT enhancer binding protein alpha (source: HGNC symbol; Acc: HGNC:1833)	5
*SLC9A3R1*	SLC9A3 regulator 1 (source: HGNC symbol; Acc: HGNC:11075)	6
*TGFBR2*	Transforming growth factor beta receptor 2 (source: HGNC symbol; Acc: HGNC:11773)	7
*LTBP1*	Latent transforming growth factor beta binding protein 1 (source: HGNC symbol; Acc: HGNC:6714)	8
*TRADD*	TNFRSF1A associated via death domain (source: HGNC symbol; Acc: HGNC:12030)	9
*SLC4A7*	Solute carrier family 4 member 7 (source: HGNC symbol; Acc: HGNC:11033)	10
*TNFRSF1B*	TNF receptor superfamily member 1B (source: HGNC symbol; Acc: HGNC:11917)	11
*LTBP4*	Latent transforming growth factor beta binding protein 4 (source: HGNC symbol; Acc: HGNC:6717)	12
*ABCB4*	ATP-binding cassette subfamily B member 4 (source: HGNC symbol; Acc: HGNC:45)	13
*MMP9*	Matrix metallopeptidase 9 (source: HGNC symbol; Acc: HGNC:7176)	14
*SLC26A8*	Solute carrier family 26 member 8 (source: HGNC symbol; Acc: HGNC:14468)	15
*TGFBR1*	Transforming growth factor beta receptor 1 (source: HGNC symbol; Acc: HGNC:11772)	16
*HIF1A*	Hypoxia-inducible factor 1 subunit alpha (source: HGNC symbol; Acc: HGNC:4910)	17
*SLC22A2*	Solute carrier family 22 member 2 (source: HGNC symbol; Acc: HGNC:10966)	18
*NOS2*	Nitric oxide synthase 2 (source: HGNC symbol; Acc: HGNC:7873)	19
*RIPK1*	Receptor-interacting serine/threonine kinase 1 (source: HGNC symbol; Acc: HGNC:10019)	20

**Table 5:** Drug–gene network analysis of pain medication.

**Gene**	**Drug**	**Indication**	**Interaction score**
*ABCB1*	Acetaminophen	Analgesic	0.004315226
*ABCB1*	Aspirin	NSAID, antithrombotic, anticoagulant	0.001591431
*ABCB1*	Capsaicin	Analgesic	0.02639903
*ABCB1*	Codeine anhydrous	Opioid, antitussive agents, analgesics	0.037398626
*ABCB1*	Gabapentin	Analgesic, for the treatment of neuropathic pain	0.016829382
*ABCB1*	Ibuprofen, sodium salt	NSAID	0.017260904
*ABCB1*	Methadone hydrochloride	Opioid, antitussive agents, analgesics	0.02639903
*ABCB1*	Naproxen sodium	NSAID, antimigraine agent	0.013199515
*ABCB1*	Pentazocine	Opioid, analgesics	0.032055965
*CFTR*	Capsaicin	Analgesic	0.070197421
*TGFB1*	Aspirin	NSAID, antithrombotic, anticoagulant	0.012623554
*TNF*	Bupivacaine	Neuralgia, analgesic, local anesthetic	0.066423367

## Data Availability

All supplementary data, code, or other materials that are related to these articles can be found publicly in an online version.

## Supporting Information

Supporting Information Additional supporting information can be found online in the Supporting Information section. File S1: The genes retrieved from GSE40445 (gene expression in versus non-CF airway epithelial cells from nasal brushing), GSE127208 (towards precision medicine for pain: diagnostic biomarkers and repurposed drugs), GSE24982 (mRNA expression profiling in the spinal nerve ligation model of neuropathic pain in rats) and GSE176223 (screening of key pathogenic genes in advanced knee osteoarthritis based on bioinformatic analysis) and the complete biochemical pathways of *TNF, ABCB1* and *TGDB1.*

## References

[1] Cystic Fibrosis Foundation (2024). 2024 Patient Registry Highlights. https://www.cff.org/medical-professionals/2024-patient-registry-highlights.

[2] Tsui L. C., Dorfman R. (2013). The Cystic Fibrosis Gene: A Molecular Genetic Perspective. *Cold Spring Harbor Perspectives in Medicine*.

[3] Paranjapye A., Ruffin M., Harris A., Corvol H. (2020). Genetic Variation in CFTR and Modifier Loci May Modulate Cystic Fibrosis Disease Severity. *Journal of Cystic Fibrosis*.

[4] Ward A., Mauleon R., Arellano J., Ooi C. Y., Rosic N. (2023). Critical Disease Burdens of Australian Adults With Cystic Fibrosis: Results From an Online Survey. *Pediatric Pulmonology*.

[5] Fillingim R. B. (2017). Individual Differences in Pain: Understanding the Mosaic That Makes Pain Personal. *Pain*.

[6] Allgood S. J., Kozachik S., Alexander K. A., Thaxton A., Vera M., Lechtzin N. (2018). Descriptions of the Pain Experience in Adults and Adolescents With Cystic Fibrosis. *Pain Management Nursing*.

[7] Collaco J. M., Cutting G. R. (2008). Update on Gene Modifiers in Cystic Fibrosis. *Current Opinion in Pulmonary Medicine*.

[8] Mésinèle J., Ruffin M., Guillot L., Corvol H. (2022). Modifier Factors of Cystic Fibrosis Phenotypes: A Focus on Modifier Genes. *International Journal of Molecular Sciences*.

[9] Ward A., Mauleon R., Ooi C. Y., Rosic N. (2024). Impact of Gene Modifiers on Cystic Fibrosis Phenotypic Profiles: A Systematic Review. *Human Mutation*.

[10] Zhou Y. H., Gallins P. J., Pace R. G. (2023). Genetic Modifiers of Cystic Fibrosis Lung Disease Severity: Whole-Genome Analysis of 7,840 Patients. *American Journal of Respiratory and Critical Care Medicine*.

[11] Bhat G. R., Sethi I., Rah B., Kumar R., Afroze D. (2022). Innovative *In Silico* Approaches for Characterization of Genes and Proteins. *Frontiers in Genetics*.

[12] Yu B. (2009). Role of In Silico Tools in Gene Discovery. *Molecular Biotechnology*.

[13] Lipner E. M., Garcia B. J., Strong M. (2016). Network Analysis of Human Genes Influencing Susceptibility to Mycobacterial Infections. *PLoS One*.

[14] Trivedi T. S., Bhadresha K. P., Patel M. P., Mankad A. U., Rawal R. M., Patel S. K. (2023). Identification of Hub Genes Associated With Human Cystic Fibrosis: A Meta-Analysis Approach. *Human Genetics*.

[15] Trouvé P., Génin E., Férec C. (2017). In Silico Search for Modifier Genes Associated With Pancreatic and Liver Disease in Cystic Fibrosis. *PLoS One*.

[16] Colli L. F. M., Cabral L. M., Matos G. C., Rodrigues C. R., de Sousa V. P. (2022). Application of In Silico Methods in Clinical Research and Development of Drugs and Their Formulation: A Scoping Review. *Journal of Applied Pharmaceutical Science*.

[17] Marques L., Costa B., Pereira M. (2024). Advancing Precision Medicine: A Review of Innovative In Silico Approaches for Drug Development, Clinical Pharmacology and Personalized Healthcare. *Pharmaceutics*.

[18] Chand T., Vaishanavaa P., Dubey A. K., Misra G. (2025). Identification of Hub Genes as Potential Diagnostic Biomarkers for Cervical Cancer: A Bioinformatic Approach. *Computational Biology and Chemistry*.

[19] George Priya Doss C., Rajasekaran R., Sudandiradoss C., Ramanathan K., Purohit R., Sethumadhavan R. (2008). A Novel Computational and Structural Analysis of nsSNPs in *CFTR* Gene. *Genomic Medicine*.

[20] Vinhoven L., Stanke F., Hafkemeyer S., Nietert M. M. (2022). Complementary Dual Approach for In Silico Target Identification of Potential Pharmaceutical Compounds in Cystic Fibrosis. *International Journal of Molecular Sciences*.

[21] Ward A., Mauleon R., Davidson G., Ooi C. Y., Rosić N. (2025). Pain in Adults With Cystic Fibrosis–Are We Painfully Unaware?. *Journal of Cystic Fibrosis*.

[22] Amberger J. S., Bocchini C. A., Schiettecatte F., Scott A. F., Hamosh A. (2015). OMIM.org: Online Mendelian Inheritance in Man (OMIM®), an Online Catalog of Human Genes and Genetic Disorders. *Nucleic Acids Research*.

[23] Li S., Brimmers A., van Boekel R. L. M., Vissers K. C. P., Coenen M. J. H. (2023). A Systematic Review of Genome-Wide Association Studies for Pain, Nociception, Neuropathy, and Pain Treatment Responses. *Pain*.

[24] Jain R., Baines A., Khan U., Wagner B. D., Sagel S. D. (2021). Evaluation of Airway and Circulating Inflammatory Biomarkers for Cystic Fibrosis Drug Development. *Journal of Cystic Fibrosis*.

[25] Giglio M., Corriero A., Preziosa A., Varrassi G., Puntillo F. (2024). The Putative Role of Immune-Inflammatory Mechanisms in Nociplastic Pain Pathways: A Narrative Review. *Exploration of Immunology*.

[26] Seychell B. C., Beck T. (2021). Molecular Basis for Protein–Protein Interactions. *Beilstein Journal of Organic Chemistry*.

[27] Yu D., Lim J., Wang X., Liang F., Xiao G. (2017). Enhanced Construction of Gene Regulatory Networks Using Hub Gene Information. *BMC Bioinformatics*.

[28] Dubin E., Lowers J., Dellon E. P. (2023). Prevalence of Unmet Pain and Symptom Management Needs in Adults With Cystic Fibrosis. *Journal of Cystic Fibrosis*.

[29] Lee A. L., Rawlings S., Bennett K. A., Armstrong D. (2016). Pain and Its Clinical Associations in Individuals With Cystic fibrosis. *Chronic Respiratory Disease*.

[30] Prajapati B. B., Filippi A., Sears E. H. (2021). Chronic Joint Pain in a Young Adult With Cystic Fibrosis. *Cureus*.

[31] Rowbotham N. J., Smith S., Leighton P. A. (2018). The Top 10 Research Priorities in Cystic Fibrosis Developed by a Partnership Between People With CF and Healthcare Providers. *Thorax*.

[32] Rowbotham N. J., Smith S., Elliott Z. C. (2023). A Refresh of the Top 10 Research Priorities in Cystic Fibrosis. *Thorax*.

[33] Jang D. I., Lee A. H., Shin H. Y. (2021). The Role of Tumor Necrosis Factor Alpha (TNF-*α*) in Autoimmune Disease and Current TNF-*α* Inhibitors in Therapeutics. *International Journal of Molecular Sciences*.

[34] Cantin A. M., Hartl D., Konstan M. W., Chmiel J. F. (2015). Inflammation in Cystic Fibrosis Lung Disease: Pathogenesis and Therapy. *Journal of Cystic Fibrosis*.

[35] Leung L., Cahill C. M. (2010). TNF-*α* and Neuropathic Pain–A Review. *Journal of Neuroinflammation*.

[36] Inglis J. J., Nissim A., Lees D. M., Hunt S. P., Chernajovsky Y., Kidd B. L. (2005). The Differential Contribution of Tumour Necrosis Factor to Thermal and Mechanical Hyperalgesia During Chronic Inflammation. *Arthritis Research & Therapy*.

[37] Lachmann H. J., Papa R., Gerhold K. (2014). The Phenotype of TNF Receptor-Associated Autoinflammatory Syndrome (TRAPS) at Presentation: A Series of 158 Cases From the Eurofever/EUROTRAPS International Registry. *Annals of the Rheumatic Diseases*.

[38] Freeman A. J., Ooi C. Y. (2017). Pancreatitis and Pancreatic Cystosis in Cystic Fibrosis. *Journal of Cystic Fibrosis*.

[39] Ooi C. Y., Dorfman R., Cipolli M. (2011). Type of CFTR Mutation Determines Risk of Pancreatitis in Patients With Cystic Fibrosis. *Gastroenterology*.

[40] Olesen S. S., Krauss T., Demir I. E. (2017). Towards a Neurobiological Understanding of Pain in Chronic Pancreatitis: Mechanisms and Implications for Treatment. *Pain Reports*.

[41] Kumar S., Ooi C. Y., Werlin S. (2016). Risk Factors Associated With Pediatric Acute Recurrent and Chronic Pancreatitis. *JAMA Pediatrics*.

[42] Guo M., Zhou X., Han X., Zhang Y., Jiang L. (2019). SPINK1 Is a Prognosis Predicting Factor of Non-Small Cell Lung Cancer and Regulates Redox Homeostasis. *Oncology Letters*.

[43] Zou W. B., Tang X. Y., Zhou D. Z. (2018). SPINK1, PRSS1, CTRC, and CFTR Genotypes Influence Disease Onset and Clinical Outcomes in Chronic Pancreatitis. *Clinical and Translational Gastroenterology*.

[44] Ziady A. G., Hansen J. (2014). Redox Balance in Cystic Fibrosis. *International Journal of Biochemistry & Cell Biology*.

[45] Németh B. C., Sahin-Tóth M. (2014). Human cationic Trypsinogen (*PRSS1*) Variants and Chronic Pancreatitis. *American Journal of Physiology-Gastrointestinal and Liver Physiology*.

[46] González-Titos A., Hernández-Camarero P., Barungi S., Marchal J. A., Kenyon J., Perán M. (2021). Trypsinogen and Chymotrypsinogen: Potent Anti-Tumor Agents. *Expert Opinion on Biological Therapy*.

[47] Beer S., Zhou J., Szabó A. (2013). Comprehensive Functional Analysis of Chymotrypsin C (CTRC) Variants Reveals Distinct Loss-of-Function Mechanisms Associated With Pancreatitis Risk. *Gut*.

[48] Derynck R., Zhang Y. E. (2003). Smad-Dependent and Smad-Independent Pathways in TGF-*β* Family Signalling. *Nature*.

[49] Snodgrass S. M., Cihil K. M., Cornuet P. K., Myerburg M. M., Swiatecka-Urban A. (2013). Tgf-*β*1 Inhibits Cftr Biogenesis and Prevents Functional Rescue of *Δ*F508-Cftr in Primary Differentiated Human Bronchial Epithelial Cells. *PLoS One*.

[50] Trojan T., Alejandre Alcazar M. A., Fink G. (2022). The Effect of TGF-*β*_1_ Polymorphisms on Pulmonary Disease Progression in Patients With Cystic Fibrosis. *BMC Pulmonary Medicine*.

[51] Liu R. M., Desai L. P. (2015). Reciprocal Regulation of TGF-*β* and Reactive Oxygen Species: A Perverse Cycle for Fibrosis. *Redox Biology*.

[52] Alkaya A., Yıldız A. E., Bağlan E., Özdel S. (2024). Unveiling the Uncommon: Diagnostic Journey of Camurati-Engelmann Disease in a Pediatric Patient. *Pediatric Rheumatology Online Journal*.

[53] Coluzzi F., Scerpa M. S., Rocco M., Fornasari D. (2022). The Impact of P-Glycoprotein on Opioid Analgesics: What’s the Real Meaning in Pain Management and Palliative Care?. *International Journal of Molecular Sciences*.

[54] Jones P. M., George A. M. (2004). The ABC Transporter Structure and Mechanism: Perspectives on Recent Research. *Cellular and Molecular Life Sciences CMLS*.

[55] Taniguchi S., Berenger F., Doi Y., Mimura A., Yamanishi Y., Okiyoneda T. (2024). Ligand-Based Virtual-Screening Identified a Novel CFTR Ligand Which Improves the Defective Cell Surface Expression of Misfolded ABC Transporters. *Frontiers in Pharmacology*.

[56] McEntire D. M., Kirkpatrick D. R., Dueck N. P. (2016). Pain Transduction: A Pharmacologic Perspective. *Expert Review of Clinical Pharmacology*.

[57] Braicu C., Buse M., Busuioc C. (2019). A Comprehensive Review on MAPK: A Promising Therapeutic Target in Cancer. *Cancers*.

[58] Zhang T., Geng M., Li X. (2024). Identification of Oxidative Stress-Related Biomarkers for Pain-Depression Comorbidity Based on Bioinformatics. *International Journal of Molecular Sciences*.

[59] Rottner M., Freyssinet J. M., Martínez M. C. (2009). Mechanisms of the Noxious Inflammatory Cycle in Cystic Fibrosis. *Respiratory Research*.

[60] Moliteo E., Sciacca M., Palmeri A. (2022). Cystic Fibrosis and Oxidative Stress: The Role of CFTR. *Molecules*.

[61] Guerini M., Condrò G., Friuli V., Maggi L., Perugini P. (2022). N-Acetylcysteine (NAC) and Its Role in Clinical Practice Management of Cystic Fibrosis (CF): A Review. *Pharmaceuticals*.

[62] Clarke L. A., Sousa L., Barreto C., Amaral M. D. (2013). Changes in Transcriptome of Native Nasal Epithelium Expressing F508del-CFTR and Intersecting Data From Comparable Studies. *Respiratory Research*.

[63] Hampton T. H., Stanton B. A. (2010). A Novel Approach to Analyze Gene Expression Data Demonstrates that the *Δ*F508 Mutation in CFTR Downregulates the Antigen Presentation Pathway. *American Journal of Physiology-Lung Cellular and Molecular Physiology*.

