# Supplementary material for: Identification of Novel Modifier Genes Associated With Pain in Cystic Fibrosis: An In Silico Gene Discovery
Source: Hum Mutat. 2025 Oct 19;2025:7570437. doi: 10.1155/humu/7570437 (PMC12554920; doi:10.1155/humu/7570437)
Supplement: Supporting Information — Additional supporting information can be found online in the Supporting Information section. File S1: The genes retrieved from GSE40445 (gene expression in versus non-CF airway epithelial cells from nasal brushing), GSE127208 (towards precision medicine for pain: diagnostic biomarkers and repurposed drugs), GSE24982 (mRNA expression profiling in the spinal nerve ligation model of neuropathic pain in rats) and GSE176223 (screening of key pathogenic genes in advanced knee osteoarthritis based on bioinformatic analysis) and the complete biochemical pathways of TNF, ABCB1 and TGDB1. [file 7570437.f1.docx]

**Table S1**. Genes retrieved from GSE40445 (Gene expression in CF-vs-non CF airway epithelial cells from nasal brushing), GSE127208 ([Towards precision medicine for pain: diagnostic biomarkers and repurposed drugs](https://www.ncbi.nlm.nih.gov/geo/query/acc.cgi?acc=GSE127208)), GSE24982 ([mRNA expression profiling in the spinal nerve ligation model of neuropathic pain in rats](https://www.ncbi.nlm.nih.gov/geo/query/acc.cgi?acc=GSE24982)) and GSE176223 (Screening of key pathogenic genes in advanced knee osteoarthritis based on bioinformatic analysis).

| GEO Accession Number | Genes |
| --- | --- |
| GSE40445 | *BANF1, EPHX1, CAB39, C2orf17, PLD3, NUCB2, SOC, PDXP, FRMD4B, ETS2, C20orf98, SLC33A1, TP53AP1, TJP1, TCEA2, AES, TETRAN, LOC348262, IFRD1, DSCR1L2, C10orf32, MGC5306, MGC20806, C16orf33, LNPEP, PHACTR2, CLDN1, RHBDL7, INSIG1, KIAA1847, ATP6V0A4, PTDSS2, DKFZP761D0211, TAF11, MRPL40, TNRC5, MAOB, KIAA1434, COBRA1, APBA2BP, GNA11, DKFZp434I1610, ARL8, SDK1, GSDMDC1, IFNGR2, TCTA, EP300, ARFGAP3, TOMM34, TTC13, HINT2, RPS6KB1, FEM1A, ERAL1, NUDT14, CCND3, RAB22A, MYCPBP, SEC14L3,CDR2, KIAA1033, RBM21, ARID1A, TTC14, ZADH1, DNCLI2, VARS2, HSPC268, TMC3, ENPP5, PBP, DREV1, C14orf31, TCERG1, CPNE2, LOC203069, B3GAT3, GSTM1, DUSP10, MB, RNF152, HSCARG, AP1G1, FLJ32096, C12orf8, LOC124446, DCTN1, APG16L, DGCR6, LOC144438, LOC90378, CUEDC2, ARRDC3, ZNF562, WDR13, EVA1, C7orf24, MECT1, FLNC, G6PC3, GRCC9, CHFR, KLF3, DKFZP564O243, ZBTB34, POLK, ENTPD4, TMC4, IFI16, PYCR2, PEX14, C6orf18, SIAT7E, UBE2M, DDX26, DKFZP586B1621, GSTM2, SP192, MGC4172, SIGIRR, CD44, FBXO28, CD19, GNB2, LOC51234, LY6D, MRPL4, NRD1, C10orf137, TSC1, PRDX5, PYCRL, PLA2G10, RABL2A, CGI-14, PIP5K3, MEA, ZNF297B, LRP16, DISP2, MLPH, TRAPPC1, HSPBP1, COG5, CREBBP, PHGDH, MGC15416, CCNE2, C6orf93, CAPS, MYO9A, KHSRP, IL12A, BDP1, FLJ13111, THAP7, LOC388389, FLJ13798, FLJ14566, TMEM1, ACO2, CUL5, LOC388962, TJP3, TPRA40, KIAA1145, NPC1, LOC389072, FLJ20406, PRNPIP, C11orf2, MGC45438, E4F1, DPM3, PTPRN2, PARD3, C12orf10, C6orf102, FLJ23342, CTSD, AP2B1, MOSPD3, CECR1, APCDD1, C6orf108, EDEM1, RUNX2, FDXR, ITGAV, SPPL2B, DHX15, GALNT7, GDI1, MID1IP1, SLC25A10, LGP1, YT521, MAP1LC3A, ALDH3B1, RALGDS, LOC285458, TFCP2L4, MLL5, IGFBP2, SMAP1, TRIM32, C20orf149, C20orf28, TGFBR2, MRPL49, RAPGEF6, DUSP8, SH3GLB1* |
| GSE127208 | *GEMIN8, MTRFR, TMEM107, MIR12136* |
| GSE24982 | *Dpp10, Htr3b, Pvalb, Mx2, Chrnb4, Rab1a, S100g, Hapln4, Vsnl1, Htr3b, Mrgprx3, Tnnc2, Kcnip4, Ano3, Sst, Mras, Tp53, Htr3a, Calml4, Ldb3, Odf3b, Mylpf, Ifitm10, Car2, Hoxd1, Spns2, Rbfox3, Tmem255a, Gabrg2, Phf24, C3, Htr1d, Rgs4, Scn11a, Gtpbp6, Vamp1, Cs, Dpysl4, Sema3f, Sorl1, Susd2, Map1b, Slc3a1, RT1-N2, Macrod1, Shank1, Dlgap3, Wbp2, Slc16a12, Slc51a, Atp1a1, Calca, Nmb, Mum1l1, Kcns1, Cdk10, Nrsn1, Perp, Slc24a2, LOC100362176, Prmt8, LOC100909866, Patj, Spock3, Nmu, Patj, Thy1, Gnmt, Slc17a7, Ttll7, Hapln1, LOC100910996, Nptx1, Kcnd3, Faim2, LOC102551017, Hs3st2, Bmp6, Hpse, Ccdc68, Tceal5, Jph3, Tfrc, Tango2, Morn5, Apitd1, Scg2, Vsnl1, Paqr5, LOC102556753, LOC100910996, Asic1, Grem2, RGD1310352, Fkbp8, Esrrg, Nfkbid, Fgf12, Insm2, Zdhhc19, Smad9, Kcnip3, Plpp4, Rgs4, Fam159b, Kcnab1, Gcnt4, Osbp2, Acot8, Ckmt2, Dgkg, Kcnip2, LOC100364027, Clec2l, Slc5a11, Kcnip4, Ntsr1, Ak1, Nlrp6, Stxbp1, Rab37, Robo2* |
| GSE176223 | *ASTN2, CALCA, CASP6, CCDC144B, CCDC85C, CCND1, CDK6, CLSPN, CNTN1, COL27A1, COL2A1, COMT, DCAF12, DENND1B, DNAJC18, EDN1, ELAC2, FAM134B, GBP1, GNG7, GSPT1, H05785, HLA-DQB1, HLA-DRB1, HRAS, Hs.554262, HTR2A, LOXL2, LY9, MBNL3, MCRS1, MFAP3, MTERF1, NF1, OSBP2, PBRM1, PHC3, PIK3CD, PNOC, PPFIBP2, PPP1R14B, PTN, RAB33A, RALGAPA2, SEPT7P2, SFPQ, SHMT1, SMURF2, SVEP1, TCF15, TNFRSF11B, TOP3A, TSPO, VEGFA, WNK1, YBX3, ZNF429, ZNF441, ZNF91, ZYX* |

**Table S2.** Biochemical pathways association of potential modifier genes in KEGG and REACTOME.

| Gene | Chemical Pathway | ID |
| --- | --- | --- |
| *TNF* | \| Adipocytokine signalling pathway \| \| --- \| \| African trypanosomiasis \| \| Allograft rejection \| \| Alzheimer's disease \| \| Amyotrophic lateral sclerosis (ALS) \| \| Antifolate resistance \| \| Antigen processing and presentation \| \| Apoptosis \| \| Asthma \| \| Death Receptor Signalling \| \| Fc epsilon RI signalling pathway \| \| Fluid shear stress and atherosclerosis \| \| Graft-versus-host disease \| \| Hematopoietic cell lineage \| \| Hepatitis C \| \| Herpes simplex infection \| \| IL-17 signalling pathway \| \| Influenza A \| \| Insulin resistance \| \| Interleukin-10 signaling \| \| Legionellosis \| \| NF-kappa B signalling pathway \| \| NOD-like receptor signalling pathway \| \| Natural killer cell-mediated cytotoxicity \| \| Pertussis \| \| RIG-I-like receptor signalling pathway \| \| Regulation of TNFR1 signalling \| \| Sphingolipid signaling pathway \| \| Systemic lupus erythematosus \| \| T cell receptor signalling pathway \| \| TNF signalling \| \| TNF signalling pathway \| \| TNFR1-induced NFkappaB signaling \| \| TNFR1-induced proapoptotic signaling \| \| TNFR1-mediated ceramide production \| \| TNFR2 non-canonical NF-kB pathway \| \| Toll-like receptor signaling pathway \| \| Type I diabetes mellitus \| \| Type II diabetes mellitus \| \| mTOR signalling pathway \| | \| KEGG:hsa04920 \| \| --- \| \| KEGG:hsa05143 \| \| KEGG:hsa05330 \| \| KEGG:hsa05010 \| \| KEGG:hsa05014 \| \| KEGG:hsa01523 \| \| KEGG:hsa04612 \| \| KEGG:hsa04210 \| \| KEGG:hsa05310 \| \| REACT:R-HSA-73887 \| \| KEGG:hsa04664 \| \| KEGG:hsa05418 \| \| KEGG:hsa05332 \| \| KEGG:hsa04640 \| \| KEGG:hsa05160 \| \| KEGG:hsa05168 \| \| KEGG:hsa04657 \| \| KEGG:hsa05164 \| \| KEGG:hsa04931 \| \| REACT:R-HSA-6783783 \| \| KEGG:hsa05134 \| \| KEGG:hsa04064 \| \| KEGG:hsa04621 \| \| KEGG:hsa04650 \| \| KEGG:hsa05133 \| \| KEGG:hsa04622 \| \| REACT:R-HSA-5357905 \| \| KEGG:hsa04071 \| \| KEGG:hsa05322 \| \| KEGG:hsa04660 \| \| REACT:R-HSA-75893 \| \| KEGG:hsa04668 \| \| REACT:R-HSA-5357956 \| \| REACT:R-HSA-5357786 \| \| REACT:R-HSA-5626978 \| \| REACT:R-HSA-5668541 \| \| KEGG:hsa04620 \| \| KEGG:hsa04940 \| \| KEGG:hsa04930 \| \| KEGG:hsa04150 \| |
| *TGFB1* | \| Cell cycle \| \| --- \| \| Cell surface interactions at the vascular wall \| \| Chronic myeloid leukemia \| \| Colorectal cancer \| \| Deubiquitination \| \| Disease \| \| Diseases of signal transduction \| \| Downregulation of TGF-beta receptor signaling \| \| ECM proteoglycans \| \| Elastic fibre formation \| \| Endocytosis \| \| Extracellular matrix organisation \| \| FoxO signaling pathway \| \| Hemostasis \| \| Hippo signaling pathway \| \| Host Interactions with Influenza Factors \| \| Infectious disease \| \| Influenza Infection \| \| Influenza Virus-Induced Apoptosis \| \| Intestinal immune network for IgA production \| \| Loss of Function of SMAD2/3 in Cancer \| \| Loss of Function of TGFBR1 in Cancer \| \| Loss of Function of TGFBR2 in Cancer \| \| Metabolism of proteins \| \| Molecules associated with elastic fibres \| \| Non-integrin membrane-ECM interactions \| \| Pancreatic cancer \| \| Pathways in cancer \| \| Platelet activation, signalling and aggregation \| \| Platelet degranulation \| \| Post-translational protein modification \| \| Renal cell carcinoma \| \| Response to elevated platelet cytosolic Ca2+ \| \| SMAD2/3 MH2 Domain Mutants in Cancer \| \| SMAD2/3 Phosphorylation Motif Mutants in Cancer \| \| Signalling by TGF-beta Receptor Complex \| \| Signalling by TGF-beta Receptor Complex in Cancer \| \| Syndecan interactions \| \| TGF-beta receptor signaling activates SMADs \| \| TGF-beta receptor signaling in EMT \| \| TGF-beta signaling \| \| TGFBR1 KD Mutants in Cancer \| \| TGFBR1 LBD Mutants in Cancer \| \| TGFBR2 Kinase Domain Mutants in Cancer \| \| TGFBR2 MSI Frameshift Mutants in Cancer \| \| Th17 cell differentiation \| \| UCH proteinases \| | \| KEGG:hsa04110 \| \| --- \| \| REACT:R-HSA-202733 \| \| KEGG:hsa05220 \| \| KEGG:hsa05210 \| \| REACT:R-HSA-5688426 \| \| REACT:R-HSA-1643685 \| \| REACT:R-HSA-5663202 \| \| REACT:R-HSA-2173788 \| \| REACT:R-HSA-3000178 \| \| REACT:R-HSA-1566948 \| \| KEGG:hsa04144 \| \| REACT:R-HSA-1474244 \| \| KEGG:hsa04068 \| \| REACT:R-HSA-109582 \| \| KEGG:hsa04390 \| \| REACT:R-HSA-168253 \| \| REACT:R-HSA-5663205 \| \| REACT:R-HSA-168254 \| \| REACT:R-HSA-168277 \| \| KEGG:hsa04672 \| \| REACT:R-HSA-3304349 \| \| REACT:R-HSA-3656534 \| \| REACT:R-HSA-3642278 \| \| REACT:R-HSA-392499 \| \| REACT:R-HSA-2129379 \| \| REACT:R-HSA-3000171 \| \| KEGG:hsa05212 \| \| KEGG:hsa05200 \| \| REACT:R-HSA-76002 \| \| REACT:R-HSA-114608 \| \| REACT:R-HSA-597592 \| \| KEGG:hsa05211 \| \| REACT:R-HSA-76005 \| \| REACT:R-HSA-3315487 \| \| REACT:R-HSA-3304356 \| \| REACT:R-HSA-170834 \| \| REACT:R-HSA-3304351 \| \| REACT:R-HSA-3000170 \| \| REACT:R-HSA-2173789 \| \| REACT:R-HSA-2173791 \| \| KEGG:hsa_M00680 \| \| REACT:R-HSA-3656532 \| \| REACT:R-HSA-3656535 \| \| REACT:R-HSA-3645790 \| \| REACT:R-HSA-3642279 \| \| KEGG:hsa04659 \| \| REACT:R-HSA-5689603 \| |
| *TNF/ TGFB1 intersect* | \| AGE-RAGE signalling pathway in diabetic complications \| \| --- \| \| Amoebiasis \| \| Chagas disease (American trypanosomiasis) \| \| Cytokine Signalling in the Immune System \| \| Cytokine-cytokine receptor interaction \| \| Developmental Biology \| \| Dilated cardiomyopathy \| \| HTLV-I infection \| \| Hepatitis B \| \| Hypertrophic cardiomyopathy (HCM) \| \| Immune System \| \| Inflammatory bowel disease (IBD) \| \| Interleukin-4 and 13 signaling \| \| Leishmaniasis \| \| MAPK signalling pathway \| \| Malaria \| \| Non-alcoholic fatty liver disease (NAFLD) \| \| Osteoclast differentiation \| \| Proteoglycans in cancer \| \| Rheumatoid arthritis \| \| Signal Transduction \| \| Signaling by Interleukins \| \| TGF-beta signaling pathway \| \| Toxoplasmosis \| \| Transcriptional regulation of white adipocyte differentiation \| \| Tuberculosis \| | \| KEGG:hsa04933 \| \| --- \| \| KEGG:hsa05146 \| \| KEGG:hsa05142 \| \| REACT:R-HSA-1280215 \| \| KEGG:hsa04060 \| \| REACT:R-HSA-1266738 \| \| KEGG:hsa05414 \| \| KEGG:hsa05166 \| \| KEGG:hsa05161 \| \| KEGG:hsa05410 \| \| REACT:R-HSA-168256 \| \| KEGG:hsa05321 \| \| REACT:R-HSA-6785807 \| \| KEGG:hsa05140 \| \| KEGG:hsa04010 \| \| KEGG:hsa05144 \| \| KEGG:hsa04932 \| \| KEGG:hsa04380 \| \| KEGG:hsa05205 \| \| KEGG:hsa05323 \| \| REACT:R-HSA-162582 \| \| REACT:R-HSA-449147 \| \| KEGG:hsa04350 \| \| KEGG:hsa05145 \| \| REACT:R-HSA-381340 \| \| KEGG:hsa05152 \| |
| *ABCB1* | \| ABC transporters \| \| --- \| \| ABC-family proteins mediated transport \| \| Abacavir transmembrane transport \| \| Abacavir transport and metabolism \| \| Bile secretion \| \| Metabolism \| \| MicroRNAs in cancer \| \| Transmembrane transport of small molecules \| | \| KEGG:hsa02010 \| \| --- \| \| REACT:R-HSA-382556 \| \| REACT:R-HSA-2161517 \| \| REACT:R-HSA-2161522 \| \| KEGG:hsa04976 \| \| REACT:R-HSA-1430728 \| \| KEGG:hsa05206 \| \| REACT:R-HSA-382551 \| |
